# Pteropod eggs released at high pCO_2_ lack resilience to ocean acidification

**DOI:** 10.1038/srep25752

**Published:** 2016-05-16

**Authors:** Clara Manno, Victoria L. Peck, Geraint A. Tarling

**Affiliations:** 1 British Antarctic Survey, Natural Environmental Research Council, High Cross, Madingley Road, Cambridge, CB3 0ET, UK

## Abstract

The effects of ocean acidification (OA) on the early recruitment of pteropods in the Scotia Sea, was investigated considering the process of spawning, quality of the spawned eggs and their capacity to develop. Maternal OA stress was induced on female pteropods (*Limacina helicina antarctica*) through exposure to present day pCO_2_ conditions and two potential future OA states (750 μatm and 1200 μatm). The eggs spawned from these females, both before and during their exposure to OA, were incubated themselves in this same range of conditions (embryonic OA stress). Maternal OA stress resulted in eggs with lower carbon content, while embryonic OA stress retarded development. The combination of maternal and embryonic OA stress reduced the percentage of eggs successfully reaching organogenesis by 80%. We propose that OA stress not only affects the somatic tissue of pteropods but also the functioning of their gonads. Corresponding *in-situ* sampling found that post-larval *L. helicina antarctica* concentrated around 600 m depth, which is deeper than previously assumed. A deeper distribution makes their exposure to waters undersaturated for aragonite more likely in the near future given that these waters are predicted to shoal from depth over the coming decades.

Early-life phases (i.e. embryogenesis and larval development) are thought to be particularly sensitive to anthropogenic environmental stressors^1^,^2^,^3^. These early stages are crucial for the viability of organisms to disperse progeny and ensure successful recruitment^4^. The survivorship of embryos and larvae underpins recruitment success^5^ and factors that increase mortality rates or delay developmental rates in these early life-phases ultimately reduce the long-term viability of populations^6^.

Ocean Acidification (OA) is one of the most serious environmental threats that we face this century. Since the beginning of the industrial revolution, the uptake of anthropogenic CO_2_ by the oceans has resulted in a decline in surface water pH and carbonate ion concentration and led to a reduction in carbonate saturation state^7^. These changes occur both from the surface layers downwards and also from the deeper layers upwards^8^ since an increase of CO_2_ to the atmosphere and surface ocean translates into a far greater change at depth^9^, due to the different geochemistry of the deeper water masses. As a result, the saturation horizon, where waters become corrosive to calcium carbonate, is predicted to shoal over the coming decades (McNeil and Matear^10^). OA is forecast to affect high-latitudes first since natural carbonate ion concentrations are already low and cold temperatures enhance the solubility of CO_2_^11^. With regards the Southern Ocean, the ecosystems most at threat are those containing regions of upwelling of nutrient depleted deep-water and the freshening of surface waters from glacial and sea ice melt, since both these factors contribute to further carbonate under-saturation^12^.

Thecosome pteropods (pelagic molluscs) have emerged as sentinel organisms in the assessment of OA’s effect on the ecosystem (ICES/OSPAR, 2015). As their thin shells are made of aragonite, a highly soluble form of biogenic CaCO_3_ in sea water^13^, pteropods are particularly vulnerable to forecasted changes in sea water carbonate chemistry^7^. Furthermore, the vertical migration behaviour of these organisms may expose them to rapid changes in the carbonate chemistry of deeper ocean layers^14^,^15^,^16^.

In the Southern Ocean, shell dissolution has already been reported in natural populations of the pteropod *Limacina helicina antarctica*^17^,^18^. In this region, pteropods comprise a significant component of the zooplankton community^19^,^20^, and are a key trophic link within Southern Ocean foodwebs^21^,^22^ as well as a major conduit for the export of carbon to the ocean interior^23^,^24^,^25^. Consequently, large decreases in pteropod populations will have significant implications for Southern Ocean biodiversity^26^ and biogeochemical-ecological processes^25^.

The reproductive characteristics of thecosome pteropods are relatively well known. The majority of species are protandrous hermaphrodites, first functioning as males and then as females^15^. The male reproductive organs degenerate as the female organs develop. The female lays fertilized, buoyant-eggs within an egg mass which consists of a sheet-like jelly mass containing transparent egg-capsules. The development of the eggs into veliger larvae is completed inside the egg-mass clutch^27^.

In benthic gastropods, OA has been shown to impact fertility and fecundity through decreasing levels of fertilization, hatching success and the survivorship of meroplanktonic larvae^28^. In pelagic molluscs, most research into OA sensitivity has otherwise concentrated on the post-larval stages, considering factors such as shell calcification and dissolution^29^,^30^,^31^, swimming behaviour^32^ and oxygen consumption^33^,^34^. The single study on post-hatch veliger larvae was performed on Mediterranean pteropods^29^, where larvae exhibited malformations and lower shell growth when exposed to OA conditions. Any impact on larval development will be severely detrimental to recruitment success and population viability, which demands a greater focus on the vulnerability of this life-stage to OA.

Here, we investigate the effect of OA on egg production and embryonic development of *Limacina helicina antarctica* in the Scotia Sea (Southern Ocean). The Scotia Sea has the largest seasonal uptake of atmospheric CO_2_ yet measured in the Southern Ocean and is a physically dynamic region where CO_2_-rich deep-water upwells to the near surface^35^,^36^,^37^. As a result, the region naturally exhibits decreased surface pH and lowered carbonate saturation, which is further augmented by anthropogenic OA^21^, making it an ideal environment in which to investigate the influence of OA on natural communities. To evaluate the impact of lowered carbonate saturation states on fecundity and embryogenic development, females and their spawned eggs were incubated in pCO_2_-manipulated seawater. This work provides an insight into the relative importance of OA-stress during the maternal-brood phase and the early developmental phase of the life-cycle. Such information enhances our ability to link the effects of OA to the recruitment potential of pteropod populations experiencing OA stress in their natural environment.

## Results

### Pteropod vertical distribution

**Figure 1:**
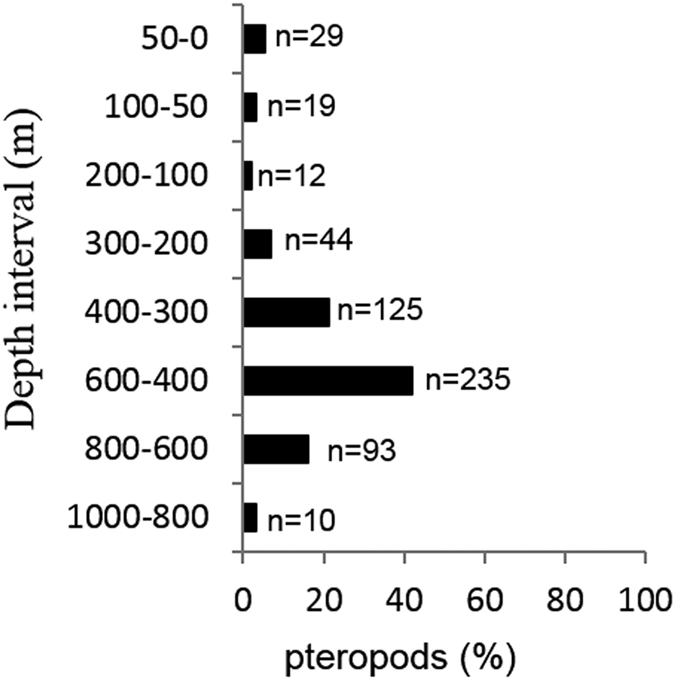
Depth-discrete distribution of post-larval pteropods. n = number of organisms captured.

The percentage of *Limacina heliciina antarctica* recovered from different water column layers is shown in Fig. 1. The plot highlights the relatively high proportion of pteropods found in the deeper-part of the water column (42% between 400–600 m; n = 235) as well as the presence of individuals (3%; n = 16) down to depths of 1000 m.

### Effect of OA on spawning and egg abundance/quality

All eggs were spherical in shape and with spherical-oval chorions (egg-capsules). They were enclosed within a gelatinous transparent mucus mass with a “bean-ribbon” shape. The eggs released on d0 and d2 had a greater intensity of yellow colour compared to those released on d5. The bean-ribbon shape of the egg masses was consistent across all egg-release events.

On d2 and d5, no eggs were laid from pteropods incubated in ambient conditions. Given that all other factors were held constant between the incubations, the results suggests that further egg releases on d2 and d5 in incubations with lowered pH levels were a maternal response to OA stress.

**Figure 2:**
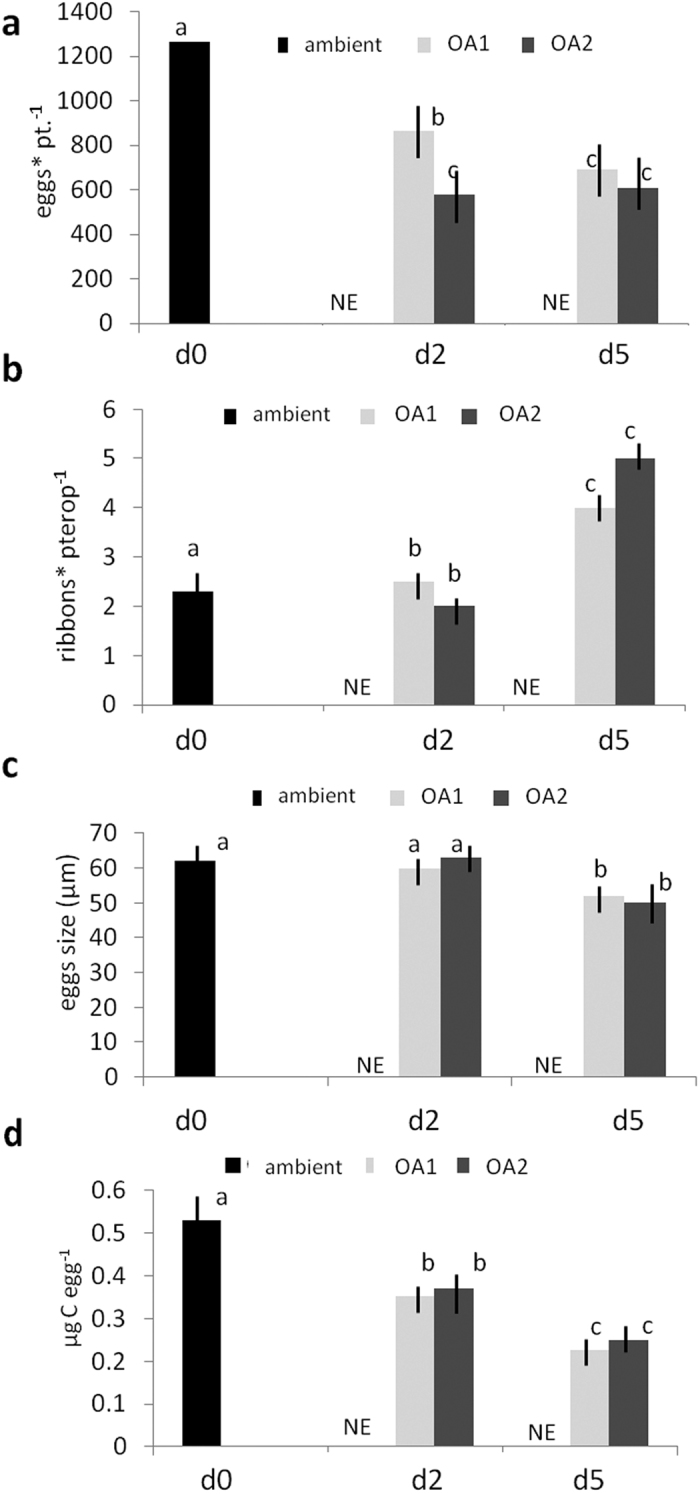
(**a**) eggs per pteropod, (**b**) ribbons per pteropod, (**c**) mean egg size (μm) and (**d**) egg-specific organic carbon content (μg C egg^−1^) at the respective egg production events d0, d2 and d5. Error bars indicate standard error of the mean. Values not sharing the same letters (located next to plotted values) are significantly different (Tukey HSD test). NE = No Eggs.

Significant variability was observed in the abundance of spawned eggs (eggs ind.^−1^) between treatments (Nested ANOVA, F = 28.90, p < 0.05 (1), df 2, 6; Fig. 2a), with eggs ind.^−1^ being significantly higher in individuals maintained in ambient conditions compared to those at lower pH (Tukey HSD, p < 0.05). Conversely significant differences were observed in the abundance of egg-mass ribbons (ribbons ind.^−1^, Fig. 2b) between the egg production events (Nested ANOVA, F = 54.47, p < 0.05 (1), df 6, 9), where ribbons ind.^−1^ were greater in the d5 egg-release event (Tukey HSD, p < 0.05). In turn, this implies that there must have been a lower number of eggs per ribbon in the d5 release event. Furthermore, the size of the eggs was significantly smaller in the d5 release event compared to the previous two release events (Nested ANOVA, F = 494.22, p < 0.05 (1), df 6, 9; Tukey HSD, p < 0.05 Fig. 2c). This pattern was also observed in the carbon content of the eggs (Nested ANOVA, F = 22.04, p < 0.05 (1), df 6, 9; Fig. 2d), which decreased significantly from the d0 to d2 release events, as well as from the d2 to the d5 events (Tukey HSD, p < 0.05).

Compared to duration of OA exposure, the intensity of OA exposure had a lesser effect on the characteristics of the spawned eggs. There was no significant difference between the size of the eggs or the ribbons ind.^−1^ between low pH (OA_1_) and extremely low pH (OA_2_) conditions. Nevertheless, OA exposure intensity did affect the eggs ind.^−1^ in the d2 release event, with egg numbers being significantly lower at OA_2_ (Nested ANOVA, F = 25, 74, p < 0.05 (1) df 6, 9; Tukey HSD, p < 0.05).

### Effect of OA on embryonic development

**Figure 3:**
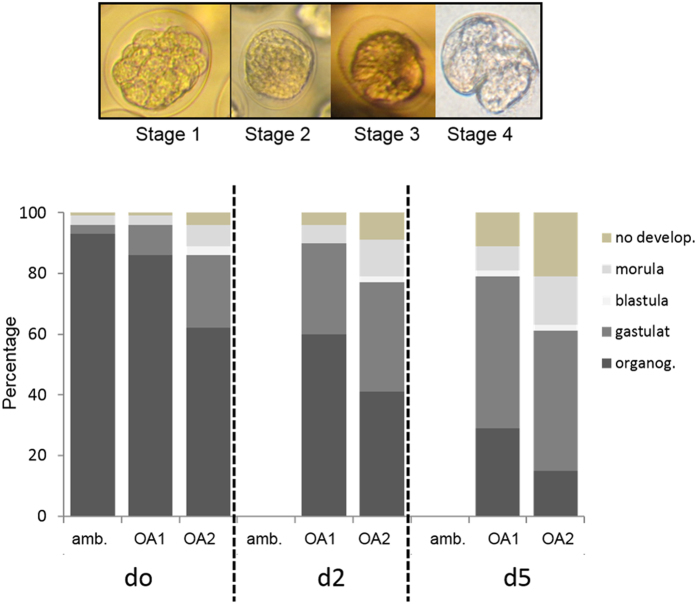
Upper - Inverted microscope images of the different egg-developmental stages. Stage 1 (morula, cleavage), Stage 2 (blastula, cleavage), Stage 3 (late gastrulation), Stage 4 (organogenesis); Lower - Embryonic development after 72 h, expressed as the percentage of the different stages at ambient conditions (amb., pH 8.), low pH (OA_1_, pH 7.8), and extremely low pH (OA_2_, pH 7.6). The eggs were incubated from females exposed to 0, 2 and 5 days of OA treatment (d0, d2 and d5 respectively).

Figure 3a shows the range of embryonic developmental stages that were observed across all experiments. The occurrence of morula-cleavage corresponds to the first stage of development, when the cells start a rapid series of divisions (stage 1). Within 72 h, the embryos develop into the following successive stages: blastula (stage 2) where the compactness of cells is lost; gastrula (stage 3), where the blastula wall undergoes invagination; and organogenesis (stage 4), where the process of organ formation begins.

Embryonic development rate was significantly affected by both the duration and intensity (Nested ANOVA, F = 22.04, p < 0.05 (1), df 6, 9) of OA exposure (Nested ANOVA, F = 14.26, p < 0.05 (1), df 2, 6) in both parents and embryos (Fig. 3b). In terms of the embryonic OA stress response, embryos exposed to lowered pH universally exhibited developmental delay. Specifically, compared to the control samples, where 95% of eggs reached the organogenesis stage within 72 h, 85% reached organogenesis at pH_1_ and 60% at pH_2_ (Tukey HSD, p < 0.05). With the addition of maternal OA stress (i.e. maternal plus embryonic OA stress), embryonic development was retarded further still, both as a factor of the duration and of the intensity of OA exposure (Tukey HSD, p < 0.05). In terms of the duration, the percentage reaching organogenesis at OA_1_ fell from 60% for d2 eggs to 30% for d5 eggs while, at OA_2_, it fell from 40% for d2 eggs to 15% for d5 eggs (Tukey HSD, p < 0.05). With regards intensity, the decrease in embryonic development was less severe, with the percentage reaching organogenesis for d2 eggs dropping from 60% at OA_1_ to 40% at OA_2_ while, for d5 eggs, the drop was from 30% to 15% respectively (Tukey HSD, p < 0.05).

## Discussion

### Effect of OA on spawning

We found that pteropods incubated in ambient seawater each spawned around 1200 eggs, with the variance between replicates being relatively small (SD ± 200). This fecundity rate appears to be higher than that reported by Paranjape^38^ at Friday Harbor, Washington, where up to 600 eggs were spawned per pteropod. Conversely, another study described the spawning of up to 5000 eggs per pteropod in Arctic waters^39^. Such high variability between pteropod spawning rates suggests that environmental conditions strongly influence fecundity in these organisms.

In our study, females started spawning within 2 h of being placed in incubation jars, during the pre-experimental acclimation period. This spawning response has also been reported in several studies^34^,^38^,^39^, which all documented spawning to take place soon after capture, even when the females were maintained in ambient seawater conditions. Even though acclimation conditions in the present study were set as close as possible to those in the natural environment, temperature changes may have been experienced during the transfer. Furthermore, handling is undoubtedly a further source of stress. Spawning may therefore be a response to these stresses. Further spawning events took place in incubations with OA manipulated conditions, but did not occur in the control incubations with ambient conditions. Exposure to OA is therefore a further stress that can induce a spawning response. We do not consider the length of the incubation to have had an adverse effect on spawning behaviour, given that the last spawn was within 5 days of capture, while Maas *et al*.^16^ and Manno *et al*.^32^ found that incubation effects on metabolic rate and behaviour were not evident until after 5 days.

The additional spawning events within the OA treatments occurred at approximately the same time across all OA treatments. This synchronisation in spawning suggests that it is the duration more than the intensity of exposure to OA conditions that induces the spawning response. Females may reach a threshold of exposure, at which point they induce spawning. The egg to ribbon ratio also decreased during these latter spawning events, with the number of ribbons per spawn increasing. A decrease in egg to ribbon ratio means that there are a greater number of egg clusters, each separate from one another and containing fewer eggs than in the initial spawn. This increase in the number of discrete ribbons is likely to result in enhanced egg dispersion, which may be beneficial in displacing the eggs away from the stressful environment to which the adult is exposed.

Studies to date suggest that *Limacina helicina* overwinters as a veliger, metamorphoses into a juvenile in spring, and increases in mass until reaching adulthood and spawning in late summer to early autumn^20^,^38^,^39^. Given that the present study was carried out in late spring to early summer, pteropods in the present study laid eggs before their natural spawning period. Pteropod eggs are described as being held within the body cavity for a considerable period in readiness for the appropriate signal to spawn^39^. Pteropods appear to retain the potential to spawn over several months, a trait that may enable them some flexibility in responding to stressful conditions.

### Effect of OA on egg quality

We found that exposure of females to lowered pH significantly reduced the number of eggs spawned per pteropod by as much as 50%. There was also a decrease in the number of eggs per ribbon, by up to 20%, and a decrease in the size and carbon content of the eggs, by up to 20% and 50% respectively. Egg production is an energetically demanding process and, under OA conditions, there may be a high energy requirement to maintain intracellular pH and to increase levels of calcification to offset shell corrosion. In this scenario, energy may be diverted away from other important physiological functions, such as egg production^40^. Nevertheless, it must also be taken into account that planktonic eggs generally decrease in size over the course of the spawning season^41^. This concurs with the present study in that it was in the last of the three spawning events that the smallest eggs were observed. Eggs from a third spawning event may have been smaller irrespective of environmental conditions, implying that the size of eggs in each of the three observed spawning events may have already been predetermined. The converse of this argument is that there was insufficient time to increase the level of resources within the third batch of eggs given that spawning was rapidly induced by the stress of OA exposure. Further experimentation is required to distinguish between these different possibilities.

A further factor to consider is that the pteropods were not fed during the incubations and consequently starvation may be an additional stressor acting in synergy with OA. A recent study found that metabolism in *L. helicina antarctica* was not significantly impacted within the first 4 days of captivity in filtered seawater^34^. This would exclude starvation as a major influence on the first two spawning events in the present study. Nevertheless, this factor may have been an additional influence on the third spawning event.

### Effect of OA on embryonic development

95% of pteropod embryos reached the organogenesis stage after 72 h when we incubated them in ambient conditions. Embryonic OA stress (i.e. exposure of eggs to OA from ambient incubated females) resulted in up to a 35% decrease in numbers reaching organogenesis compared to the controls (those eggs maintained in ambient conditions). However, the combination of maternal and embryonic OA stress resulted in up to an 80% decrease in the number of eggs reaching organogenesis. Consequently, we can assume that maternal stress accounts for more than a halving of successful development in the resulting embryos. This finding highlights the high degree of sensitivity of pre-spawn life stages to OA. Their smaller carbon content means that eggs produced under OA conditions have decreased levels of energetic reserves to facilitate successful development.

The synergistic impact of maternal- and embryonic OA stress best represents the situation in the natural environment where pteropods become exposed to OA at all stages of their life cycle. Previous studies on juvenile and adult pteropods incubated in OA conditions found relatively high levels of survivorship (up to 80% over scales of weeks to months)^31^,^32^,^42^. Here we show that survivorship under the same OA scenarios is considerably lower in the very early life stages.

The capacity of the capsule surrounding the embryos to buffer the decrease in pH in pteropod eggs is still unknown. However, the fact that we observed a negative impact on embryonic development over all OA treatments suggests that capsules mainly provide mechanical protection for the eggs. This is in agreement with Pechenik *et al*. (1999)^4^ who considered egg encapsulation in meroplanktonic molluscs. Noisette *et al*.^43^ found that gastropod embryos were affected by high pCO_2_ during intracapsular development, but removal of the egg capsule resulted in more severe OA impacts on embryonic development. The permeability and capacity for acidosis in the intracapsular fluid surrounding the eggs needs to be further explored to determine the extent to which it can buffer against OA stress.

### Pteropod vertical distribution and consequences for recruitment success

We found that the majority of the pteropod population occurred within the mesopelagic layers, mainly between 400 and 600 m, but down to the maximum sampled depth interval of 800 to 1000 m. These organisms therefore appear to undertake extensive vertical migrations in the Southern Ocean. Seasonal migrations into the deeper, colder layers may be a means of lowering metabolic rate^14^,^44^,^45^ and could be used as an overwintering strategy to minimise energy expenditure at a time of year when food resources are low^14^. However, our observations were made in the late spring to early summer period when overwintering had most likely ceased. The deep distribution of the pteropods may therefore be a result of daily vertical migrations between the surface layer at night and deeper layers during the daytime. Such a large migration range suggests that these pteropods have prolific swimming capabilities.

In the present study region of the Scotia Sea, the lysocline, (the aragonite compensation depth where Ω_A_ values approach 1) is located at around 1000 m throughout most of this region^37^. The lysocline is shoaling through oceanic absorption of anthropogenic OA^46^ and is predicted to reach the surface layers (in winter) by 2030. Reduced ventilation further elevates CO_2_ concentrations at depth, because the decline in O_2_ is accompanied by a corresponding accumulation of respiratory CO_2_^9^. Therefore, the fact that females are distributed deep in the water column means they will be exposed to undersaturated waters much sooner than if they mainly occupied the surface layers.

We found that it was the duration of exposure more than the intensity of the exposure of females to OA that had the greatest impact on spawning and egg quality and that even an exposure of 2 days was sufficient to reduce fecundity and the ability for successful embryonic development. The exposure time of pteropods to low pH waters during their diel and seasonal migrations is then a crucial factor on which greater focus is required. Pteropods may also pass through eddy structures containing water upwelled from deeper layers, which are undersaturated for aragonite^26^. Such encounters with low pH, undersaturated water will become more frequent and extended as anthropogenic CO_2_ levels continue to rise. Better parameterisation of the OA exposure duration against levels of fecundity and developmental success is therefore a major requirement to consider the full impact of OA on the viability of pteropod populations in the Southern Ocean.

This study has shown female exposure to OA conditions negatively impacts both fecundity and embryonic development. It suggests that declines in the level of pteropod recruitment may occur in advance of the lysocline reaching the surface layers, and could be seen even before 2030.

## Methods

**Figure 4:**
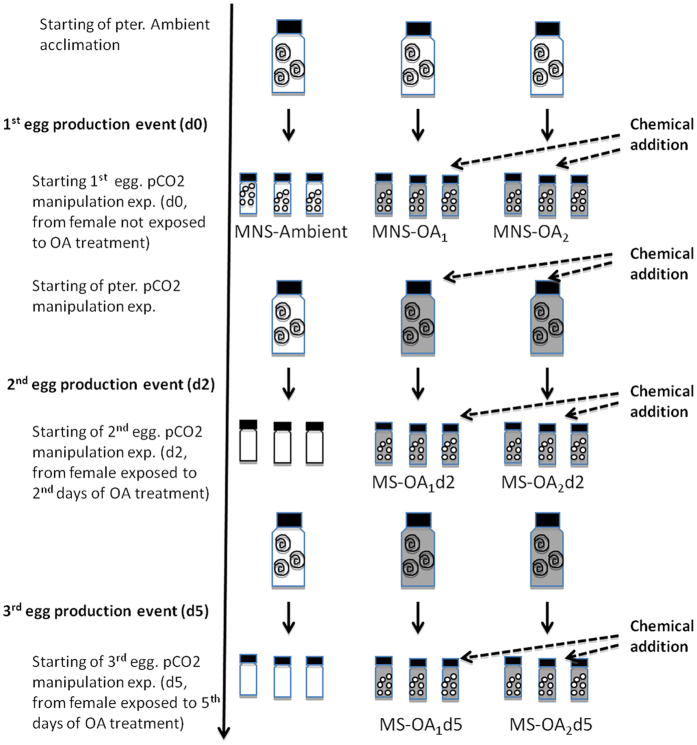
Schematic representation of the experimental design. Note that empty vials signify that no eggs were spawned. MS: maternally stressed eggs; MNS: maternally non-stressed eggs; MS-OAd2: maternally stressed eggs exposed to OA conditions for 2 d; MS-OAd5: maternally stressed eggs exposed to OA conditions for 5 d. Subscripts 1 and 2 relate to manipulated pH conditions of 7.8 and 7.6 respectively.

Pteropods (*Limacina helicina antarctica*) were collected by a motion-compensated Bongo net (200 μm mesh) and a MOCNESS net (333 μm mesh) in December 2013, in the Scotia Sea, (55.246°S and 41.265°W, 3200 m bottom-depth) on board the *RRS James Clark Ross* (Cruise number JR291; Fig. 4). The Bongo net was deployed between the surface and a maximum depth of 50 m, while the MOCNESS sampled a series of 8 different depth intervals (1000–800, 800–600, 600–400, 400–300, 300–200, 200–100, 100–50, 50–0 m). Mean water column salinity and temperature between 0 and 50 m were 34.1 and 1.9 °C respectively. Specimens used for incubation experiments were recovered from the Bongo net deployments only, after gently removing them from the cod-ends. MOCNESS samples were immediately preserved in 90% buffered ethanol for subsequent assessment of vertical distribution and abundance at the home laboratory.

### Experiment design

Live pteropods were isolated and acclimatised within 0.2 μm filtered seawater at ambient temperature (~2 °C) for 24 h. During acclimatisation, T and pH of the incubation water was checked regularly. Post-acclimitisation, females were identified following the description of Lalli and Gilmer^15^ and separated out from the other specimens. 500 ml plastic incubation jars were filled with manipulated seawater (see below) using a plastic tube (extending from a master tank to the bottom of the incubation jar) to minimize exchange of CO_2_ with air. Out of all the pteropods collected, we found a total of 9 ovigerous females which we carefully transferred by pipette into 3 incubation jars (3 individuals per jar), which was subsequently sealed without any air in the headspace to limit the exchange of CO_2_ with the atmosphere. The experiment was run at ambient *in situ* temperature (~2 °C) through immersion of the jars in a temperature-controlled water bath and in constant darkness.

#### Adult incubation and spawning

Incubations were run at 3 different pH levels: 8.0 (ambient conditions), 7.8 (low pH, OA_1_), and 7.6 (extremely low pH, OA_2_). Respectively, these three levels represent pCO_2_ levels that reflect present day conditions (387 μatm) and two potential future states (750 μatm and 1200 μatm). The pteropods were incubated for 8 days in each of the experiments. pH, T and pteropod survivorship were monitored daily. All pteropods at the end of experiment were alive (100% survival) and in a good state of health (i.e. as indicated by persistent upward swimming). Egg production was monitored every 2 h during the incubations. In total, there were three egg production events: during the period of acclimation in ambient seawater (i.e. within the first 24 h, d0) and on day 2 (d2) and day 5 (d5) of the incubation experiment (Fig. 4). To avoid egg cannibalism and degradation, a plastic pipette was used to remove eggs from the incubation bottle within 2 h of them being laid. The incubation bottles were covered with parafilm during this operation to minimise the exchange of CO_2_ with the atmosphere, with the plastic pipette accessing the bottle through a small hole in the parafilm. Half of the eggs harvested from each egg production event were used in separate manipulation experiments (see below), with the other half being preserved in buffered ethanol for analyses of abundance and carbon content. At the end of the incubation period, the adult specimens were carefully recovered and either preserved or frozen.

#### Incubation of eggs

Harvested eggs were immediately introduced into 100 ml vials containing seawater from either ambient or manipulated sources, as used for the adult incubations (i.e. ambient, OA_1_ and OA_2_ respectively). Each of the egg-incubations were performed in triplicate. In total, this amounted to a total of 81 separate jars of eggs (triplicate samples of 3 egg production events from 3 jars of pteropods manipulated in 3 different sets of seawater conditions). The jars were incubated for a total of 72 h at ambient seawater temperature (~2 °C) and the eggs then preserved in buffered ethanol for analysis at the home laboratory.

#### Synthesis of experimental strategy

In the above experimental design, eggs were produced from females before and during their exposure to manipulated OA conditions (Fig. 4). Those eggs that were spawned during pre-OA exposure were then incubated in ambient, low or extremely low pH conditions for 72 h. Eggs spawned during OA exposure were subsequently incubated in low pH (OA_1_) or extremely low pH (OA_2_). The design gave us the opportunity to compare the influence of OA exposure during the embryonic development phase alone (embryonic OA stress), and to the influence of OA stress during both the egg-brood phase and embryonic development phase (maternal plus embryonic OA stress). Comparing the performance of eggs spawned at d0 across the 3 different OA regimes investigated embryonic OA stress, while that of eggs spawned during d2 and d5 and then incubated at either low or extremely pH levels investigated combined maternal and embryonic OA stress.

We confirms that all experimental protocols were approved by a named institutional committee and that the methods were carried out in accordance with the approved guidelines.

### Manipulation of chemistry parameters

#### Acid-base additions

Seawater chemistry was manipulated through adding a combination of Na_2_CO_3_ and acid (HCl) to alter pH while maintaining total alkalinity (TA). The acid and base additions were calculated using *seacarb* software^47^. This calculation relies on knowledge of the TA, Dissolved Inorganic Carbon concentration (DIC) and pH of the seawater, although one of these three parameters can be estimated if values for the other two are known. In this instance, we based the calculation on values determined for pH and TA. pH was measured directly using a pH electrode (Metrohm 826, MERK standards solution, NBS). TA was determined through applying a surface-salinity and -temperature based algorithm, based on Lee *et al*.^48^, and refined through spatially-intensive carbonate chemistry surveys in the region:


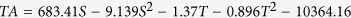


where S is surface-salinity and T is surface-temperature in °C (M.P. Humphreys, pers. comm.)

After acid and base additions, the pH of the manipulated seawater was measured every 24 h to assess variability over the course of the incubations.

#### Carbonate-chemistry analyses

At the start and end of each incubation experiment, sub-samples of seawater were fixed with 2% mercuric chloride for subsequent shore-based carbonate chemistry analyses. The TA and DIC of each sample was measured simultaneously by a potentiometer titration system using a technique based on the method of Edmond^49^, with a closed cell described by Goyet *et al*.^50^. The accuracy (3 mmol kg^−1^ for TA and 4 mmol kg^1^ for DIC) was determined by analysing Certified Reference Material (CRM) with known TA and DIC concentrations. Carbonate saturation state with respect to aragonite (Ωa) was indirectly calculated from TA and DIC data using the CO_2_ SYS software^51^ with carbonate dissociation constants by Mehrbach *et al*.^52^ refitted by Dickson and Millero^53^ and sulphate dissociation constants by Dickson^54^. The carbonate system parameters of seawater cultures and calculated Ωa at the beginning and the end of the incubation experiments are given in Table 1.

### Analyses of eggs and embryos

A Leica M165 stereomicroscope was used to count and measure the eggs as well as to estimate the total area of the mucus mass. For Carbon (C) content, the sacs were dissected and the eggs placed on Whatman GF/F filters previously combusted for 4 h at 550 °C to eliminate any residual carbon. CHN analysis was performed by a CE-440 Elemental Analyser (Exeter Analytical, 285 Inc.). Average carbon content per egg was calculated by dividing total carbon by total egg volume. Total egg volume was estimated geometrically through assuming each egg was a sphere.

The developmental stage of the embryos was determined through observations made under an inverted Olympus microscope. Morphological analyses determined the percentage of eggs that were at the cleavage and gastrulation developmental stages, based on Raven *et al*.^55^.

### Data analysis

We performed a nested (hierarchical) analysis of variance (ANOVA) within one main factor (Zar 1996) to determine whether differences in experimental treatment (i.e. exposure to pH_1_ or pH_2_) resulted in significant differences in rates of development (% reaching the organogenesis stage), the number of eggs produced, egg size and egg carbon-content. Nested ANOVA tests were employed because the eggs used in the experiments came from three different batches, where the females responsible for each batch were unknown and random. Accordingly, the experiment had a hierarchy, with two pH treatments being the ‘samples’, and the eggs batches, the ‘sub-samples’. A nested ANOVA accounts for the within-group variability in the sub-samples (egg batches), so enabling hypotheses regarding the samples (pH treatments) to be appropriately addressed. Values were considered significant at the p < 0.05 level. Tukey’s honest significant difference test (HSD) was used to check for differences between groups. Differences were considered significant at p < 0.05. HSD tests were performed by Statistica (Windows, version 6.0). All data were pre-checked for normality and variance homogeneity (Levene’s test).

## Additional Information

**How to cite this article**: Manno, C. *et al*. Pteropod eggs released at high pCO_2_ lack resilience to ocean acidification. *Sci. Rep.* **6**, 25752; doi: 10.1038/srep25752 (2016).

## Figures and Tables

**Table 1:** Mean values of carbonate system parameters of sea water cultures at the start and end of the perturbation experiments: T (C), pH, TA (Total alkalinity, mmol kg^−1^), TC (Total CO_2_ mmol kg^−1^), pCO_2_ (Partial pressure of CO_2_), Ωa (Aragonite saturation state), S (Salinity).

	T (C)	S	PH	TA	TC	pCO_2_	Ωa
Control	1.9 ± 0.52	34.1 ± 0.02	8.1 ± 0.02	2330.32 ± 10.5	2218.31 ± 17	375 ± 14	1.42 ± 0.21
PH1(OA1)	1.9 ± 0.54	34.1 ± 0.02	7.8 ± 0.01	2330.32 ± 8.90	2281.14 ± 06	750 ± 24	0.91 ± 0.14
PH2(OA2)	1.9 ± 0.54	34.1 ± 0.02	7.6 ± 0.02	2330.32 ± 9.50	2299.01 ± 11	1200 ± 34	0.76 ± 0.05

The pH treatments refer to target pCO_2_ values (386 ppm, 750 ppm and 1100 ppm). Note: pH and T were monitored each day (SD pH < 0.03; SD T < 0.15).
